# A Study of the Essential Oil Isolated from *Ageratina dendroides* (Spreng.) R.M. King & H. Rob.: Chemical Composition, Enantiomeric Distribution, and Antimicrobial, Antioxidant, and Anticholinesterase Activities

**DOI:** 10.3390/plants12152796

**Published:** 2023-07-28

**Authors:** Eduardo Valarezo, Emmily Jaramillo-Jaramillo, Ana Carrión-Campoverde, Vladimir Morocho, Ximena Jaramillo-Fierro, Luis Cartuche, Miguel Angel Meneses

**Affiliations:** Departamento de Química, Universidad Técnica Particular de Loja, Loja 110107, Ecuador; ecjaramillo4@utpl.edu.ec (E.J.-J.); abcarrion1@utpl.edu.ec (A.C.-C.); svmorocho@utpl.edu.ec (V.M.); xvjaramillo@utpl.edu.ec (X.J.-F.); lecartuche@utpl.edu.ec (L.C.); mameneses@utpl.edu.ec (M.A.M.)

**Keywords:** *Eupatorium dendroides*, *Raulinoreitzia crenulata*, chiral compounds, andro encecalinol, germacrene D

## Abstract

*Ageratina dendroides* is an aromatic species native to Ecuador. In this study, the chemical composition, enantiomeric distribution, and antifungal, antibacterial, antioxidant, and anticholinesterase activities of the essential oil isolated from aerial parts of *Ageratina dendroides* were determined. The quantitative chemical composition was determined using gas chromatography equipped with a flame ionization detector. The qualitative chemical composition was determined using gas chromatography coupled with mass spectrometry. The enantiomeric distribution was determined using an enantioselective chromatographic column. A broth microdilution method was used to determine the antibacterial activity. This antibacterial activity was tested against three Gram-negative bacilli bacteria and three Gram-positive cocci bacteria. The antifungal activity was tested against two fungi, a fungus, and a yeast. The antioxidant activity was determined using the ABTS (2,2-azino-bis(3-ethylbenzthiazoline-6-sulfonic acid)) method and DPPH (2,2-diphenyl-1-picrylhydrazyl) method. The anticholinesterase activity was analyzed using the spectrophotometric method. Sixty-eight volatile compounds were identified in the essential oil. These compounds represent 99.49% of the total composition. In terms of the number of compounds and their relative abundance, the most representative group was sesquiterpene hydrocarbons, with thirty-four compounds and an abundance of 49.22%. The main constituents were found to be andro encecalinol (14.41 ± 1.26%), germacrene D (8.86 ± 1.06%), amorpha-4,9-dien-14-al (7.68 ± 0.70%), *β*-sesquiphellandrene (7.01 ± 1.49%), *α*-muurolol (5.89 ± 0.93%), and 7-*epi*-*α*-selinene (5.68 ± 0.53%). Five pairs of enantiomers were identified in the essential oil of *Ageratina dendroides*. The essential oil did not report antimicrobial activity at the maximum concentration tested (MIC > 4000 µg/mL) against any of the microorganisms tested. The antioxidant activity of the essential oil was strong in the ABTS method, with an SC_50_ of 33.2 ± 1.4 µg/mL. Additionally, the *Ageratina dendroides* essential oil reported moderate anticholinesterase activity, with an IC_50_ of 297.8 ± 1.03 µg/mL.

## 1. Introduction

The Asteraceae family is one of the largest and most diverse families, including about 1778 genera and 33,994 species worldwide [1]. This family is characterized by its compound flowers, which are made up of multiple small flowers grouped together in a structure called a capitulum. The Asteraceae family is known for its wide therapeutic use and deep-rooted presence in traditional medicine. Some of its members have been cultivated for more than 3000 years, both for food and medicinal purposes [2]. Although they are more common in the arid and semi-arid regions of subtropical areas, they are known and distributed throughout the world. Members of the Asteraceae family exhibit a wide range of beneficial activities, including anti-inflammatory, antimicrobial, antioxidant, and hepatoprotective properties [3]. In addition, in traditional medicine, this family of plants has been used as an anti-inflammatory, antipyretic, diaphoretic in fevers, hepatoprotective, astringent, muscle relaxant, nervous tonic, and laxative, and to treat bleeding, wounds, headaches, spasmodic diseases, flatulence, haemorrhoids, gangrenous ulcer, dysentery, dyspepsia, leucorrhoea, lumbago, and disorders causing cachexia [2]. Phytochemical studies carried out on the Asteraceae family have shown that many components of this family have remarkable biological activity. Some of these compounds include flavonoids, phenolic acids, coumarins, terpenoids (such as monoterpenes, sesquiterpenes, diterpenes, and triterpenes), and sterols. The diversity of the compounds in this plant family makes it an interesting source for phytochemical research and the search for new compounds with potential therapeutic applications [4]. Within the Asteraceae family, *Ageratina* is an American genus of the tribe Eupatorieae (subfamily Asteroideae), which comprises about 327 species [5].

In Ecuador, the Asteraceae family is widely represented by more than 200 genera and plays a crucial role in the plant diversity of the country. One of the most important genera of this family in Ecuador is *Ageratina*. This genus includes a variety of shrubby and herbaceous species that are distributed in different regions of the country [6]. An important species of *Ageratina* in Ecuador is *Ageratina dendroides* (Spreng.) R.M. King & H. Rob (syn. *Eupatorium dendroides* Spreng. and Raulinoreitzia crenulata (Spreng.) R.M. King & H. Rob.). This endemic species, also known as “chilca” or “chilca de cerro”, grows in the mountainous areas of Ecuador between 1500 and 3500 m a.s.l., mainly in the provinces of Azuay, Cañar, Loja, and Zamora Chinchiple [7]. *Ageratina dendroides* is also a species native to the tropical regions of Central and South America, where it is found mainly in areas of humid jungle and tropical forests, both in lowlands and at higher altitudes [8].

*Ageratina dendroides* is a perennial shrub that reaches a height of approximately 1 to 2 m. It has a woody and branched stem, with a light brown bark. The leaves are simple, opposite, and lanceolate or oval in shape, with toothed or serrated edges. The surface of the leaves is smooth and shiny, and their color can vary between dark green and light green. The inflorescences of *Ageratina dendroides* appear in the form of terminal racemes composed of numerous small flowers. Each flower is surrounded by narrow bracts and possesses a tubular corolla composed of fused petals, which can be white, cream, or pale pink in color. The fruit is a small, dry achene containing a single seed [8]. The species *Ageratina dendroides* plays an important ecological role by providing food and shelter for a variety of pollinators, such as bees and butterflies. Their presence in ecosystems contributes to the pollination of other plant species and the conservation of biological diversity [9]. *Ageratina dendroides* has aroused the interest of the scientific community due to its possible medicinal properties. This plant species has been traditionally used in Ecuadorian folk medicine to cure blows and infections [10]. However, despite the promising characteristics of *Ageratina dendroides*, the scientific research on this species has been limited to date.

Studies carried out on alcoholic and hexanic extracts from species of the genus *Ageratina* have reported flavonoids, flavones, and flavanones as the main constituents that could have therapeutic applications in modern medicine [11,12]. Likewise, studies carried out with essential oils (Eos) obtained from the genus *Ageratina* have demonstrated the presence of several compounds, including carvacrol, spathulenol, terpinen-4-ol, thymol, nerodiol, cubebene, *β*-caryophyllene, *β*-myrcene, *α*-pinene, limonene, pentacosane, 8,9-epoxythymyl isobutyrate, germacrene D, thymyl isobutyrate, eupatoriochromene, and encecalol [13,14,15]. On the other hand, studies carried out with extracts and essential oils obtained from species of the genus *Ageratina* have revealed a variety of biological activities, including repellent, antiviral, antibacterial, and cytotoxic activity [16]. However, to date, there is no specific scientific information on the chemical composition, enantiomeric distribution, and biological activity of the essential oils of *Ageratina dendroides*. Therefore, in this scientific article, it is intended to shed light on the chemical nature of the volatile compounds that make up the essential oil (EO) of *Ageratina dendroides* and the biological activity it possesses. This knowledge will allow for a determination of the scientific potential and possible applications of this essential oil in the field of medicine, as well as in the conservation of biodiversity through knowledge of this.

## 2. Results

### 2.1. Essential Oil Isolated

An approximate total of 15 kg (divided into three distillations) of fresh plant material (with a moisture of 63.5 ± 1.2% *w*/*w*) from aerial parts from *Ageratina dendroides* were hydrodistilled to isolate the EO. About 4.5 mL of EO was obtained from this vegetal material, which represents a yield of 0.03 ± 0.01% (*v*/*w*) or 0.3 ± 0.1 mL/kg.

### 2.2. Physical Properties of Essential Oil

The EO from *Ageratina dendroides* was presented as an unctuous liquid less dense than water. Table 1 shows the mean values and standard deviations (SD) of the physical properties of the EO.

### 2.3. Chemical Composition of Essential Oil

The identification and quantification of the volatile compounds were carried out using gas chromatography coupled with mass spectrometry (GC-MS) and gas chromatography coupled with a flame ionization detector (GC-FID). Table 2 shows the relative abundance (%) with the standard deviation (SD), retention time (RT), chemical formula (CF), monoisotopic mass (MM), and retention indices calculated (RIC) and obtained from the literature (RIL) for each compound. Sixty-nine volatile compounds were identified in the EO of *Ageratina dendroides*. These compounds represent 99.49% of its total composition. The compounds were classified according to their chemical nature (number of carbons and presence of oxygen), and as a result, it was obtained that the compounds were found within four groups. These were a group of monoterpenes (10 carbons) that were non-oxygenated (MH), two groups of sesquiterpenes (15 carbons): oxygenated (OS) and non-oxygenated (SH), and one group of other compounds (OC, non-terpenic compounds). In terms of the number of compounds and their relative abundance, the most representative group was SH, with thirty-five compounds and an abundance of 49.22%. In fact, three of the six main compounds belonged to this group. The presence of oxygenated monoterpenes and diterpenes (oxygenated and non-oxygenated) was not determined. The main constituents (>5%) were found to be OC andro encecalinol (CN: 63, CF: C_14_H_16_O_2_, MM: 216.11 Da) with an abundance of 14.41 ± 1.26%, SH germacrene D (CN: 24, CF: C_15_H_24_, MM: 204.19 Da) with 8.86 ± 1.06%, *β*-sesquiphellandrene (CN: 38) with 7.01 ± 1.49%, 7-*epi*-*α*-selinene (CN: 37) with 5.68 ± 0.53%, OS amorpha-4,9-dien-14-al (CN: 68, CF: C_15_H_22_O, MM: 218.17 Da) with 7.68 ± 0.70%, and *α*-muurolol (CN: 59, CF: C_15_H_26_O, MM: 222.20 Da) with 5.89 ± 0.93%.

### 2.4. Enantiomeric Analysis

Five pairs of enantiomers were separated in the *Ageratina dendroides* EO using a column with an enantioselective stationary phase. The retention time (RT), retention indices (RI), enantiomeric distribution (ED), and enantiomeric excess (e.e.) for each pair of compounds are shown in Table 3. The (+)-*γ*-cadinene and (−)-*γ*-cadinene were found to be practically like a racemic mixture (racemate) with an e.e. of 4.05%.

### 2.5. Antimicrobial Activity

The antibacterial and antifungal activities of the EO from *Ageratina dendroides* were evaluated using the microdilution broth method. A total of eight microorganisms were evaluated: three Gram-negative bacilli, three Gram-positive cocci, a fungus, and a yeast. The tested microorganisms and the values of the minimum inhibitory concentration (MIC) of the *Ageratina dendroides* EO, positive control, and negative control are shown in Table 4. It was not possible to determine the MIC value of the *Ageratina dendroides* EO against any of the microorganisms at the maximum concentration tested (400 µg/mL). All the microorganisms showed normal growth in the negative control. 

### 2.6. Antioxidant Activity 

The ABTS and DPPH methods were used to determine the antioxidant activity of the *Ageratina dendroides* EO. The ABTS method was based on the scavenging capacity of the EO against ion radical 2,2′-azino-bis(3-ethylbenzothiazoline-6-sulfonic acid) (ABTS^•+^) and in the DPPH method, the EO’s scavenging capacity was determined against radical 2,2-diphenyl-1-picrylhydrazyl (DPPH^•^). The scavenging capacity (SC_50_, µg/mL) of the EO and the positive control values with their respective standard deviations (SD) are shown in Table 5. 

### 2.7. Anticholinesterase Activity

**Figure 1:**
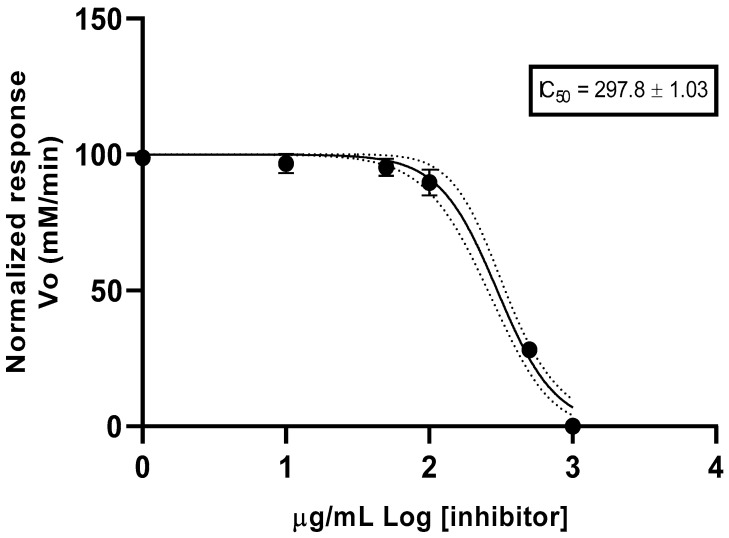
Anticholinesterase activity of essential oil from *Ageratina dendroides*.

The spectrophotometric method was used to determine the anticholinesterase (anti-AChE) activity. The IC_50_ (half-maximal inhibitory concentration) value was measured from the corresponding rate of the reaction curve (Figure 1). The EO exhibited an IC_50_ value of 297.8 ± 1.03 µg/mL and donepezil (positive control) reported an IC_50_ value of 12.40 ± 1.35 µg/mL.

## 3. Discussion

The yield of the *Ageratina dendroides* EO was 0.3 mL/kg, which can be considered as low according to the classification (values of< 5 mL/kg are considered to be low, between 5 mL/kg and 10 mL/kg are considered to be intermediate, and values of >10 mL/kg are considered to be high) given by the categorization proposed by the Agency of Ciencia y Tecnología para el Desarrollo (CYTED) [17]. The EO yields from the leaves of *Ageratina jahnii* (B.L. Rob.) R. M. King & H. Rob. and *Ageratina pichinchensis* (Kunth) R. M. King & H. Rob collected in Mérida, Venezuela, were 0.50% and 0.43% (*w*/*v*), respectively [14]. In the present study, sixty-nine volatile compounds were identified, representing more than 99% of the total composition of the essential oil from *Ageratina dendroides*. The compounds were classified into four groups according to their chemical nature. Non-oxygenated sesquiterpenes were the most representative group, with thirty-five compounds and an abundance of 49.22%. Previous studies carried out by Valarezo et al. in 2021 on the chemical composition of the EO of the leaves of *Ageratina dendroides* showed the presence of forty-five individual compounds, representing more than 99% of the total composition of the EO; in this studio, nineteen compounds belonging to the SH group were identified, which represented 62.35% [15]. The main constituents in the EO of the leaves of *A. dendroides* were SH germacrene D (29.92 ± 0.68%), *δ*-cadinene (9.31 ± 0.11%), and *cis*-cadine-1,4-diene (5.48 ± 0.04%) [15]. In the present study, these compounds were found, but with different percentages; these differences could be due to the different phenological state of the species at the time of collecting the plant material, since the chemical composition of an essential oil can be affected by extrinsic (shade, rain, humidity, soil, and others) and intrinsic (phenological state, age of the plant, and part of the plant used, among others) factors [18]. 

Within the genus Ageratina, the existence of EO has been reported in some of its species. In the EO from the leaves of *Ageratina jahnii*, fifteen components were identified and the major components were *β*-myrcene (37.6%), *α*-pinene (17.1%), limonene (8.8%), and pentacosane (9.2%). In the EO of *A. pichinchensis*, twenty-five components were identified, the main ones being 8,9-epoxythymyl isobutyrate (20.2%), germacrene D (19.8%), thymyl isobutyrate (10.8%), eupatoriochromene (6.5%), and encecalol (5.9%) [14]. Carvacrol was the main compound in the EO of Mexican *A. jocotepecana* extracted from its leaves (30.08%) and flowers (32.5%) [13]. Seventy components were identified in the EO from *A. adenophora* and the main constituents were *γ*-muurolene (16.88%), o-cymene (11.17%), bornyl acetate (8.62%), and *α*-bisabolol (7.12%) [19]. In the EO from the leaves of *A. pentlandiana* grown in Cuzco (Peru), one hundred and fifteen compounds were identified and the main components were 2,5-dimethoxy-p-cymene (20.9%), thymol methyl ether (9.9%), and 2,5-dimethoxy-p-cymenene (8.2%) [20].

This is the first report of an enantioselective GC-MS analysis of the EO from *Ageratina dendroides*, and this analysis showed the presented five pairs of enantiomeric compounds, including (+/−)-*α*-copaene, (+/−)-epizonarene, (+/−)-*γ*-cadinene, (+/−)-bicyclogermacrene, and (+/−)-*α*-muurolol. The ability to separate these enantiomers indicates the presence of chiral compounds in the essential oil. The enantiomeric ratio of an essential oil is important information, which could be related to the biological activity, metabolism, and organoleptic quality of the enantiomeric pairs [21]. The (+)-*γ*-cadinene and (−)-*γ*-cadinene were found almost as a racemic mixture or racemate (e.e. 4.05%), which could have important biological implications [2]. Indeed, racemic mixtures can influence the organoleptic properties and interactions with biological receptors, which may have consequences in terms of biological activity and therapeutic potential [3]. Despite the lack of complete separation in the case of (+)-*γ*-cadinene and (−)-*γ*-cadinene, the obtained results still provided valuable information on the chemical composition of the essential oil of *Ageratina dendroides*. The successful identification and separation of the other pairs of enantiomers demonstrated the complexity of the compounds present in the EO and their potential chemical diversity.

Regarding its antimicrobial activity, the EO from *Ageratina dendroides* was inactive against the microorganisms evaluated at the maximum concentration of 4000 µg/mL. To the best of our knowledge, this is the first report on the antimicrobial activity of the EO from *A. dendroides*. In 2017, Van Vuuren and Holl established a criterium for the classification of the antimicrobial activity for extracts and essential oils, and in this sense, values of >1001 µg/mL are considered to be inactive [22]. As a comparison, the antimicrobial results for Ageratina species were presented. Torres-Barajas et al., in 2013, evaluated the antibacterial activity of the Eos from *Ageratina jahnii* and *Ageratina pichinchensis* using the disc diffusion agar method and reported activity against *Staphylococcus aureus* and *Enterococcus faecalis*, with MIC values of 49.5 mg/mL for *A. jahnii* and 104 mg/mL for *A. pichinchensis* [14]. These values are higher than 4000 µg/mL, however, the methods were different, where microdilution was performed in a liquid state while the disc diffusion agar in a solid state, and this difference influenced the rate of diffusion for the EO in the microorganism medium. Solis-Quispe et al., in 2019, using the disc diffusion agar method, reported strong antibacterial activity of the EO from *Ageratina pentlandiana* against *S. aureus* (MIC 11.9 µL/mL), *Bacillus subtilis* (22.7 µL/mL), *E. coli* (64.8 µL/mL), and *Salmonella thipymurium* (50 µL/mL) [20]. However, the antibacterial activities were different due to the variation in the chemical composition.

The antioxidant activity of the EO from *A. dendroides* showed different values for the ABTS (SC_50_ 33.2 µg/mL) and DPPH (SC_50_ 4586.2 µg/mL) assays. The lower activity with the DPPH method may be explained by the low capacity of the non-oxygenated terpenes from donating a hydrogen atom [23]. According to Anthony et al. (2012), who studied different essential oils and their antioxidant activity, the authors proposed that an EO with SC_50_ values less than 300 µg/mL could be considered as exceptionally active [24]. Vasanthi and Gopalakrishnan, in 2013, reported the antioxidant activity for the EO from *Ageratina adenophora* (Spreng) as SC_50_ for ABTS and DPPH assays with values of 71.25 µg/mL and 60.2 µg/mL, respectively [25]. The determination of antioxidant properties allows for an evaluation of the effectivity of an EO in radical-mediated process; EOs are known to scavenge free radicals, and in vitro antioxidant assays represent the first attempt to propose their use for health benefits.

The scientific evidence about using EOs in the treatment of Alzheimer’s disease has motivated the evaluation of the anti-AChE of volatile fractions of aromatic plants. Benny and Thomas, in 2018, summarized the significant effects of EOs on modulating pathologies through different mechanisms, including anticholinesterase activity [26]. This is the first report of the anti-AChE activity for the EO from *Ageratina dendroides*, with an IC_50_ value of 297.8 µg/mL. This activity could be considered as being of moderate potency (20 < IC_50_ < 200 µg/mL), according to the scale proposed by Santos et al. in 2018 [27]. Other studies have reported the anti-AChE for the EO from *Ageratina adenophora* (Vasanthi and Gopalakrishnan, 2013), with an IC_50_ of 92.25 µg/mL [25]. The complexity of the chemical composition of essential oils is associated with these different values. 

## 4. Materials and Methods

### 4.1. Materials

Acetylthiocholine (AcSCh), acetylcholinesterase (AChE), butylated hydroxytoluene (BHT), dimethyl sulfoxide (DMSO), dichloromethane (DCM), donepezil, magnesium chloride hexahydrate, methanol (MeOH), phosphate-buffered saline (PBS), sodium sulfate anhydrous, tris hydrochloride (Tris-HCl), trolox, 2,2′-azinobis-3-ethylbenzothiazoline-6-sulfonic acid (ABTS), 2,2-diphenyl-1-picrylhydryl (DPPH), and 5,5′-dithiobis (2-nitrobenzoic acid) (DTNB) were purchased from Sigma-Aldrich (San Luis, MO, USA). Fluid thioglycollate medium, Mueller-Hinton II broth, Mueller-Himton broth, and Sabouraud dextrose broth were purchased from DIPCO (Quito, Ecuador). Helium was purchased from INDURA (Quito, Ecuador). The standard aliphatic hydrocarbons were purchased from ChemService (West Chester, PA, USA). All the chemicals were of analytical grade and used without further purification.

### 4.2. Plant Material

The aerial parts (leaves and flowers) of *Ageratina dendroides* were collected on the Villonaco hill in the canton and province of Loja. The collection was carried out in a place located at 3°59′38.3″ south longitude and 79°15′49.4″ west latitude at an altitude of 2700 m a.s.l. After being collected, the plant material was stored and transferred in airtight plastic containers. Botanist Vladimir Morocho made the identification of the plant material. A voucher specimen was deposited at the Herbarium of Universidad Técnica Particular de Loja (HUTPL).

### 4.3. Postharvest Treatments 

Once the material arrived at the laboratory, one hour after being collected, the post-harvest treatment was carried out, which included the elimination of foreign or degraded plant material.

### 4.4. Moisture Determination

The moisture of the plant material was determined using the method of Loss on drying (Moisture) in plants (AOAC 930.04-1930), according to Equation (1). For this, an analytical balance (Mettler AC 100, Mettler Toledo, Columbus, OH, USA) was used.

(1) $$Moisture\left(\%\right)=\frac{wi-wo}{wi}*100$$

where w is the weight sample of “i” initial and “o” after drying.

### 4.5. Essential Oil Isolation

The extraction of the EO was carried out using hydrodistillation in Clevenger-type apparatus (80 L distiller). Initially, 16 L of water was placed in the distiller, then the plant material, and the extraction process began. The process was carried out for 3 h, counted from the fall of the first drop of distillate. The obtained steam (EO and water) was condensed and the essential oil was separated via decantation. Anhydrous sodium sulfate was used to dry the EO. Once dry, the essential oil was stored at 4 °C in amber sealed vials.

### 4.6. Determination of the Physical Properties of the Essential Oil

The density of the essential oil was determined using the ISO 279:1998 standard [28] (equivalent to the AFNOR NF T 75-111 standard), the refractive index (index of refraction) was determined using the ISO 280:1998 standard [29] (similar to AFNOR NF T 75-112), and the optical rotation was determined according to the ISO 592:1998 standard [30]. An analytical balance (Mettler AC 100, Mettler Toledo, Columbus, OH, USA) was used to determine the density, a refractometer (model ABBE, BOECO, Hamburg, Germany) to determine the refractive index, and an automatic polarimeter (Mrc-P810, MRC, Holon, Israel) to determine the optical rotation. The subjective color was obtained online, for which a photograph taken of the EO with a white background was uploaded to the PINETOOL website https://pinetools.com/ (accessed on 20 February 2023). All the measurements were taken at 20 °C.

### 4.7. Essential Oil Compounds Identification

#### 4.7.1. Quantitative Analysis

The quantitative analysis was performed using gas chromatography coupled with a flame ionization detector (GC-FID), for which a Thermo Scientific gas chromatography (Trace 1310, Waltham, MA, USA), a flame ionization detector (FID), a nonpolar GC column (DB-5ms, stationary phase 5%-phenyl-methylpolyxilosane, 30 m of length, 0.25 mm of diameter, and 0.25 µm of stationary layer thickness), and an automatic injector (AI 1310, Thermo Scientific, Waltham, MA, USA) were used. For the sample preparation, 1 µL of solution (1/100, *v*/*v*, EO/DCM) was injected, with a split ratio of 1:50. Helium was used as a carrier gas at 1 mL/min in constant flow mode with an average velocity of 25 cm/s. The injector and detector temperatures were 230 °C. The oven temperature program included an initial isotherm of 50 °C for 3 min, followed by a temperature ramp to 230 °C at 3 °C/min (60 min), and a final isotherm for 3 min (total run time 66 min). The relative amounts of the compounds were calculated based on the GC peak area (FID response), without using a correction factor.

#### 4.7.2. Qualitative Analysis

The qualitative analysis was performed using gas chromatography coupled with mass spectrometry (GC-MS), for which the same equipment was used as that in the quantitative analysis, except for the detector, which was replaced by a mass spectrometer (MS) (quadrupole) detector (ISQ 7000, Thermo Scientific, Waltham, MA, USA). The sample concentrations and temperatures (ramp, injector, and detector) were the same as those in qualitative analyses. Helium was used as a carrier gas at 0.9 mL/min in constant flow mode with an average velocity of 34 cm/s. The operating conditions for the MS were as follows: electron multiplier 1600 eV, 70 eV, mass range 40–350 *m*/*z*, and scan rate 2 scan/s. Equation (2) [31] was used to determine the retention index (RI) of each compound. For the identification of the compounds, the IR and mass spectra were compared with published data [32].

(2) $$RI=100C+100\frac{\left(RTx-RTn\right)}{\left(RTN-RTn\right)}$$

where C is the carbon number of the aliphatic hydrocarbons (C_9_ to C_25_) that elute before the compound of interest. RT is the retention time of x compound of interest, n is the aliphatic hydrocarbons that elute before of the compound of interest, and N is the hydrocarbons that elute after of the compound of interest.

### 4.8. Antimicrobial Activity

#### 4.8.1. Antibacterial Activity

The antibacterial activity of the *Ageratina dendroides* EO was tested against five strains of bacteria, two Gram-negative bacilli bacteria: *Escherichia coli* O157:H7 (ATCC 43888) and *Pseudomonas aeruginosa* (ATCC 10145), and three Gram-positive cocci bacteria: *Enterococcus faecium* (ATCC 27270), *Enterococcus faecalis* (ATCC 19433), and *Staphylococcus aureus* (ATCC 25923). The procedures were performed as previously described by Valarezo et al., 2021 [33]. Briefly, the antibacterial assay was developed into a 96 microwell plate according to the microdilution broth method. Two-fold serial dilution was used to obtain a concentration of the EO ranging from 4000 to 15.62 µg/mL. Ciprofloxacin was used as a positive control for *Escherichia coli* and *Pseudomonas aeruginosa,* and ampicillin for *Enterococcus faecium*, *Enterococcus faecalis*, and *Staphylococcus aureus*. The maximum evaluated concentration was 4000 µg/mL and DMSO at 5% was used as a negative control. The minimum inhibitory concentration (MIC), the lowest concentration of an antimicrobial that inhibits the growth of a microorganism after its incubation, was used to report the activity values.

#### 4.8.2. Antifungal Activity

The antifungal activity of the *Ageratina dendroides* EO was tested against two strains of fungi, a fungus: *Aspergillus niger* (ATCC 6275), and a yeast: *Candida albicans* (ATTC 10231). The procedures were performed as previously described by Valarezo et al., 2021 [33]. Briefly, the MIC was determined using a final concentration of 5 × 104 spores/mL in 96 microwell plates. The EO was dissolved in Sabouraud dextrose broth with a fungal inoculum to achieve the required concentrations from 4000 to 15.62 µg/mL. Ciprofloxacin was used as a positive control and DMSO as a negative control. 

### 4.9. Evaluation of Antioxidant Capacity 

#### 4.9.1. ABTS Radical Cation Scavenging Activity

The ABTS methods were used to determine the free radical scavenging activity of the *Ageratina dendroides* EO. For this, the reagent 2,2′-azino-bis (3-ethylbenzothiazoline-6-sulfonic acid) was used to produce 2,2′-azino-bis(3-ethylbenzothiazoline-6-sulfonic acid) radical cation (ABTS^•+^). The procedures were carried out according to those described by Valarezo et al., 2021 [34]. Briefly, the antiradical capacity of the EO was assessed against ABTS^•+^ by measuring the rate of reduction at 734 nm, using a UV spectrophotometer (Genesys 10S UV-Vis Spectrophotometer, Thermo Fisher Scientific, Waltham, MA, USA). Stabilized radicals in methanol, adjusted to an optical density of 1.1 ± 0.02, were mixed with different concentrations of the EO at room temperature for one hour. The antiradical capacity was expressed as half scavenging capacity (SC_50_), calculated from the corresponding curve fitting. Trolox was used as a positive and MeOH as a negative control. The maximum evaluated concentration was 2000 µg/mL.

#### 4.9.2. DPPH Radical Scavenging Activity

The free radical scavenging activity of the *Ageratina dendroides* EO was also determined using the DPPH method. In the DPPH method, the reagent 2,2-diphenyl-1-picrylhydrazyl was used to produce 2,2-diphenyl-1-picrylhydrazyl radical (DPPH^•^). The procedures were carried out according to those described by Valarezo et al., 2021 [34]. Briefly, the antiradical capacity of the EO was assessed against DPPH^•^ by measuring the rate of reduction at 515 nm. The equipment, the positive and negative control, and the maximum concentration evaluated were the same as those for the ABTS radical cation scavenging activity.

### 4.10. Anticholinesterase Activity

An anticholinesterase assay was performed according to the principle described by Ellman et al. [35], according to the procedure described by Valarezo et al., 2021 [36], with slight modifications. The mix of the reaction, containing buffer Tris 50 mM pH 8.0, acetylthiocholine (15 mM), Ellman’s reagent DTNB (3 mM), and the EO at different decreasing concentrations, was pre-incubated at 25 °C for three minutes. Later, acetylcholinesterase from electric eels (0.5 U/mL) was added to start the reaction and the progression was monitored at 412 nm in a microplate spectrophotometer (EPOCH 2, BioTek, Winooski, VT, USA). The half inhibitory concentration (IC_50_) was extracted from the non-linear regression model (normalized response vs. log Inhibitor-variable slope). MeOH was used as a negative control and donepezil hydrochloride as a positive control.

### 4.11. Statistical Analysis

All the procedures were performed in triplicate, except for the identification of the essential oil compounds, enantioselective analysis, and antimicrobial activity, which were performed nine times. The data were collected in a Microsoft Excel sheet. The statistical software Minitab 17 (Version 17.1.0., Minitab LLC., State College, PA, USA) was used to calculate the measures of the central tendency and standard deviation. The data from the antiradical assays were analyzed using the GraphPad Prism, version 6.0 software (GraphPad Software Inc., San Diego, CA, USA). The data from the anticholinesterase assays were analyzed using the GraphPad Prism, version 6.0 software (GraphPad Software Inc., San Diego, CA, USA).

## 5. Conclusions

The enantiomeric distribution, antibacterial activity, antifungal activity, antioxidant capacity, and anticholinesterase activity of the essential oil from *Ageratina dendroides* were determined for the first time. Sixty-eight chemical compounds were identified, with andro encecalinol being the main compound. The *Ageratina dendroides* essential oil exhibited exceptional antioxidant activity and moderate anticholinesterase activity. With this research, we contribute to the knowledge on the endemic aromatic plants of Ecuador. This study lays the foundation for future research on the biological properties, aroma profiles, and potential applications of the enantiomers identified in the essential oil of *Ageratina dendroides*. For future studies, it is proposed to investigate the anti-inflammatory activity of this essential oil.

## Figures and Tables

**Table 1:** Physical properties of the essential oil of *Ageratina dendroides*.

	*Ageratina dendroides* EO	
	Mean	SD
Density, ρ (g/cm^3^)	0.8816	0.0001
Refractive index, *n*^20^	1.5001	0.0016
Specific rotation, [α] (°)	+44.6	3.6
Subjective color	light yellow	
RGB color values	R: 247, G: 240, B: 25	
CMYK color values	C: 0, M: 3, Y: 90, K: 3	
Hex Color Codes	#f7f019	

**Table 2:** Chemical composition of essential oil from *Ageratina dendroides*.

CN	RT	Compound	RIC	RIR	%	SD	Type	CF	MM (Da)
1	11.29	*ο*-Cymene	1024	1022	0.51	0.07	MH	C_10_H_14_	134.11
2	24.36	Thymol, methyl ether	1235	1232	0.33	0.05	OC	C_11_H_16_O	164.12
3	27.69	*neoiso*-Verbanol acetate	1329	1328	0.18	0.01	OC	C_12_H_20_O_2_	196.15
4	30.25	*δ*-Elemene	1333	1335	2.16	0.09	SH	C_15_H_24_	204.19
5	30.86	*α*-Cubebene	1344	1348	0.48	0.07	SH	C_15_H_24_	204.19
6	31.99	Neryl acetate	1364	1359	0.06	0.00	OC	C_12_H_20_O_2_	196.15
7	32.36	Cyclosativene	1366	1369	0.20	0.01	SH	C_15_H_24_	204.19
8	33.15	*α*-Copaene	1373	1374	0.23	0.03	SH	C_15_H_24_	204.19
9	34.38	*β*-Cubebene	1385	1387	0.48	0.11	SH	C_15_H_24_	204.19
10	34.54	*β*-Elemene	1388	1389	0.19	0.01	SH	C_15_H_24_	204.19
11	34.77	*α*-Cedrene	1413	1410	0.15	0.01	SH	C_15_H_24_	204.19
12	35.83	*β*-Funebrene	1416	1413	0.41	0.01	SH	C_15_H_24_	204.19
13	35.96	(*E*)-Caryophyllene	1417	1417	4.45	0.70	SH	C_15_H_24_	204.19
14	36.26	*β*-Cedrene	1422	1419	0.16	0.02	SH	C_15_H_24_	204.19
15	36.38	*β*-Gurjunene	1428	1431	0.22	0.01	SH	C_15_H_24_	204.19
16	37.05	6,9-Guaiadiene	1439	1442	0.35	0.08	SH	C_15_H_24_	204.19
17	37.25	(*E*)-Cinnamyl acetate	1444	1443	0.43	0.06	OC	C_11_H_12_O_2_	176.08
18	37.43	Amorpha-4,11-diene	1447	1449	0.48	0.03	SH	C_15_H_24_	204.19
19	37.47	*α*-Humulene	1454	1452	1.07	0.14	SH	C_15_H_24_	204.19
20	37.59	*α*-Acoradiene	1458	1464	0.51	0.12	SH	C_15_H_24_	204.19
21	37.74	Dauca-5,8-diene	1471	1471	0.64	0.08	SH	C_15_H_24_	204.19
22	37.84	trans-Cadina-1(6),4-diene	1476	1475	2.42	0.62	SH	C_15_H_24_	204.19
23	37.95	*γ*-Muurolene	1479	1478	0.82	0.12	SH	C_15_H_24_	204.19
24	38.12	Germacrene D	1482	1480	8.86	1.09	SH	C_15_H_24_	204.19
25	38.21	*γ*-Himachalene	1483	1481	1.83	0.13	SH	C_15_H_24_	204.19
26	38.30	*δ*-Selinene	1486	1492	0.47	0.04	SH	C_15_H_24_	204.19
27	38.45	*trans*-Muurola-4(14),5-diene	1491	1493	0.78	0.08	SH	C_15_H_24_	204.19
28	38.69	*α*-Zingiberene	1495	1493	0.51	0.08	SH	C_15_H_24_	204.19
29	38.79	*epi*-Cubebol	1496	1493	2.39	0.76	OS	C_15_H_26_O	222.20
30	38.92	Bicyclogermacrene	1498	1500	0.70	0.13	SH	C_15_H_24_	204.19
31	39.07	Epizonarene	1502	1501	0.44	0.02	SH	C_15_H_24_	204.19
32	39.15	*β*-Bisabolene	1508	1505	2.87	0.80	SH	C_15_H_24_	204.19
33	39.40	(*Z*)-*γ*-Bisabolene	1510	1506	0.33	0.08	SH	C_15_H_24_	204.19
34	39.75	*γ*-Cadinene	1513	1513	0.32	0.04	SH	C_15_H_24_	204.19
35	39.94	Cubebol	1517	1514	0.43	0.02	OS	C_15_H_26_O	222.20
36	40.08	7-*epi*-*α*-Selinene	1518	1520	5.68	0.53	SH	C_15_H_24_	204.19
37	40.20	*β*-Sesquiphellandrene	1525	1521	7.01	1.49	SH	C_15_H_24_	204.19
38	40.39	<*γ*->Vetivenene	1533	1531	1.25	0.21	SH	C_15_H_22_	202.17
39	40.83	*trans*-Cadina-1,4-diene	1541	1533	0.61	0.15	SH	C_15_H_24_	204.19
40	41.00	*β*-Vetivenene	1548	1554	0.63	0.11	SH	C_15_H_22_	202.17
41	41.16	Germacrene B	1559	1559	1.51	0.12	SH	C_15_H_24_	204.19
42	41.90	*E*-Nerolidol	1563	1561	0.37	0.13	OS	C_15_H_26_O	222.20
43	42.06	Spathulenol	1577	1577	0.54	0.04	OS	C_15_H_24_O	220.18
44	42.78	Caryophyllene oxide	1585	1582	0.40	0.14	OS	C_15_H_24_O	220.18
45	42.89	Globulol	1589	1590	0.11	0.01	OS	C_15_H_26_O	222.20
46	43.00	Viridiflorol	1592	1592	1.09	0.11	OS	C_15_H_26_O	222.20
47	43.14	Carotol	1595	1594	0.03	0.00	OS	C_15_H_26_O	222.20
48	43.30	Guaiol	1599	1600	0.06	0.00	OS	C_15_H_26_O	222.20
49	43.44	Geranyl 2-methyl butanoate	1602	1601	0.30	0.01	OS	C_15_H_26_O_2_	238.19
50	43.54	1,10-di-*epi*-Cubenol	1614	1618	1.62	0.15	OS	C_15_H_26_O	222.20
51	43.63	10-*epi*-*γ*-Eudesmol	1619	1622	0.46	0.04	OS	C_15_H_26_O	222.20
52	43.75	1-*epi*-Cubenol	1627	1627	0.54	0.02	OS	C_15_H_26_O	222.20
53	43.94	*γ*-Eudesmol	1628	1630	0.89	0.10	OS	C_15_H_26_O	222.20
54	44.08	*cis*-Cadin-4-en-7-ol	1631	1632	2.00	0.45	OS	C_15_H_26_O	222.20
55	44.14	*β*-Acorenol	1634	1635	0.43	0.01	OS	C_15_H_26_O	222.20
56	44.26	*epi*-*α*-Cadinol	1637	1636	1.34	0.09	OS	C_15_H_26_O	222.20
57	44.35	*β*-Acorenol	1638	1638	0.14	0.01	OS	C_15_H_26_O	222.20
58	44.43	*α*-Muurolol (=Torreyol)	1643	1644	5.89	0.93	OS	C_15_H_26_O	222.20
59	44.63	Cubenol	1649	1645	0.63	0.04	OS	C_15_H_26_O	222.20
60	44.75	7-*epi*-*α*-Eudesmol	1663	1662	0.63	0.04	OS	C_15_H_26_O	222.20
61	44.85	Bulnesol	1671	1670	0.51	0.20	OS	C_15_H_26_O	222.20
62	44.98	Andro encecalinol	1674	1675	14.41	1.26	OC	C_14_H_16_O_2_	216.11
63	45.11	Khusinol	1678	1679	0.53	0.09	OS	C_15_H_24_O	220.18
64	45.20	*α*-Bisabolol	1692	1685	3.34	0.34	OS	C_15_H_26_O	222.20
65	45.34	Cyperotundone	1696	1695	0.21	0.01	OS	C_15_H_22_O	218.17
66	45.64	Amorpha-4,9-dien-2-ol	1701	1700	1.20	0.16	OS	C_15_H_24_O	220.18
67	45.88	Amorpha-4,9-dien-14-al	1702	1704	7.68	0.70	OS	C_15_H_22_O	218.17
68	57.08	n-Tetracosane	2401	2400	0.59	0.15	OC	C_24_H_50_	309.03
		Monoterpene hydrocarbons			0.51				
		Sesquiterpene hydrocarbons			49.22				
		Oxygenated sesquiterpene			33.76				
		Other compounds			16.00				
		Total identified			99.49				

CN: compound number, assigned according to their elution order; RT retention time; RIC: calculated retention indices; RIR: literature retention indices; %: relative abundance; CF: chemical formula; MM: monoisotopic mass; SD: standard deviation; and Tr: traces.

**Table 3:** Chiral compounds present in the essential oil of *Ageratina dendroides*.

RT	Enantiomers	RI	ED (%)	e.e. (%)
26.79	(+/−)-*α*-Copaene	1375	24.89	50.23
27.08		1380	75.11	
33.59	(+/−)-Epizonarene	1489	18.05	63.89
33.88		1494	81.95	
35.60	(+/−)-*γ*-Cadinene	1524	52.03	4.05
35.71		1526	47.97	
35.92	(+/−)-Bicyclogermacrene	1629	43.00	13.99
36.02		1731	57.00	
49.20	(+/−)-*α*-Muurolol	1769	83.13	66.25
49.40		1773	16.87	

**Table 4:** Antimicrobial activity of essential oil from *Ageratina dendroides*.

Microorganism	*Ageratina dendroides*	Positive Control	Negative Control
	MIC (µg/mL)		
Gram-negative bacilli			
*Escherichia coli* O157:H7 (ATCC 43888)	>4000	1.56	+
*Pseudomonas aeruginosa* (ATCC 10145)	>4000	0.39	+
*Salmonella enterica* subs enterica serovar Thypimurium WDCM 00031, derived (ATCC 14028)	>4000	0.39	+
Gram-positive cocci			
*Enterococcus faecium* (ATCC 27270)	>4000	0.39	+
*Enterococcus faecalis* (ATCC 19433)	>4000	0.78	+
*Staphylococcus aureus* (ATCC 25923)	>4000	0.39	+
Fungi and yeasts			
*Aspergillus niger* (ATCC 6275)	>4000	0.10	+
*Candida albicans* (ATTC 10231)	>4000	0.10	+

+: normal growth.

**Table 5:** Antioxidant activity of essential oil from *Ageratina dendroides*.

Sample	ABTS	DPPH
	SC_50_ (µg/mL) ± SD	
*Ageratina dendroides* EO	33.2 ± 1.4	4586.2 ± 1.1
Trolox	29.1 ± 1.1	35.54 ± 1.1

## Data Availability

Data are available from the authors upon reasonable request.
